# Unifying global efforts by CEPI’s centralized laboratory network

**DOI:** 10.3389/fimmu.2024.1404309

**Published:** 2024-04-11

**Authors:** Ali Azizi, Valentina Bernasconi

**Affiliations:** ^1^ Laboratory Research and Innovations (LRI), CEPI, Washington DC, United States; ^2^ Laboratory Research and Innovations (LRI), CEPI, Oslo, Norway

**Keywords:** vaccine, assays, global health, pandemic, network

The Coalition for Epidemic Preparedness Innovations (CEPI) established the Centralized Laboratory Network (CLN) in March 2020 in response to the SARS-CoV-2 pandemic. Initially, the network comprised a few high-quality laboratories located in North America, Europe, and Asia (1, 2). CEPI’s primary goal was to ensure that all transferred assays to these facilities used the same methods, materials, reference standards, and controls to standardize vaccine evaluation. This approach aimed to streamline and harmonize the assessment of vaccine candidates, enabling more efficient and reliable comparisons between different vaccines under development.

During the SARS-CoV-2 pandemic, the CLN played a crucial role by offering free testing of all samples for vaccine developers, regardless of their size, location and funding status (2, 3). This commitment to providing free testing ensured that developers, especially those with limited resources, could access the necessary services to advance their SARS-CoV-2 vaccine candidates.

After our significant contribution to controlling the SARS-CoV-2 pandemic, the CLN has expanded significantly, becoming the largest global network conducting global vaccine testing with 17 laboratories from 13 different countries announced so far. To date, five members of the CLN are in Low- and Middle-income Countries (LMICs) including Bangladesh, India, Kenya, Senegal, and Uganda, indicating an expansion in scale and geographical reach into the Global South. This expansion strengthens CEPI-CLN’s ability to standardize and accelerate the evaluation of vaccines against epidemic and pandemic diseases. By expanding into the Global South, the CLN reduces sample transfer and testing times, potentially accelerating vaccine development against emerging infectious diseases. In addition, we ensure that vaccine developers in each geographic region have access to the same level of resources. CEPI-CLN is poised to continue its vital role in accelerating the development of vaccines against emerging infectious diseases. Our network currently standardizes the assessment of vaccine efficacy against rare but potential diseases including Lassa, Nipah, Monkeypox, Sudan, Marburg, MERS, and others, ensuring uniformity and accelerating standardized assays for vaccines.

Looking ahead, CEPI remains committed to supporting vaccine developers. For funded CEPI developers, testing of samples will continue to be provided free of charge and for non-funded developers, CEPI will offer testing services at an affordable cost, ensuring that financial constraints do not hinder the progress of potential vaccine candidates. This commitment underscores CEPI’s mission to support global health by facilitating the development of vaccines against epidemic and pandemic diseases. In addition to providing free or affordable testing services, CEPI is committed to transparency and collaboration. All protocols and standard procedures within the CLN will be made accessible to anyone who requires them. This transparency not only fosters trust and collaboration within the scientific community but also ensures that best practices are shared and implemented across the network.

## Author contributions

AA: Writing – original draft, Methodology, Supervision, Validation. VB: Writing – review & editing, Methodology, Supervision, Validation.
